# The genetic spectrum of Gitelman(-like) syndromes

**DOI:** 10.1097/MNH.0000000000000818

**Published:** 2022-07-11

**Authors:** Karl P. Schlingmann, Jeroen H.F. de Baaij

**Affiliations:** a Department of General Pediatrics, University Children's Hospital, Münster, Germany; b Department of Physiology, Radboud Institute for Molecular Life Sciences, Radboud University Medical Center, Nijmegen, The Netherlands

**Keywords:** Gitelman syndrome, mitochondria, Na^+^-Cl^−^-co-transporter, salt-wasting, tubulopathy

## Abstract

Gitelman syndrome is a recessive salt-wasting disorder characterized by hypomagnesemia, hypokalemia, metabolic alkalosis and hypocalciuria. The majority of patients are explained by mutations and deletions in the *SLC12A3* gene, encoding the Na^+^-Cl^−^-co-transporter (NCC). Recently, additional genetic causes of Gitelman-like syndromes have been identified that should be considered in genetic screening. This review aims to provide a comprehensive overview of the clinical, genetic and mechanistic aspects of Gitelman(-like) syndromes.

**Recent findings:**

Disturbed Na^+^ reabsorption in the distal convoluted tubule (DCT) is associated with hypomagnesemia and hypokalemic alkalosis. In Gitelman syndrome, loss-of-function mutations in *SLC12A3* cause impaired NCC-mediated Na^+^ reabsorption. In addition, patients with mutations in *CLCKNB*, *KCNJ10*, *FXYD2* or *HNF1B* may present with a similar phenotype, as these mutations indirectly reduce NCC activity. Furthermore, genetic investigations of patients with Na^+^-wasting tubulopathy have resulted in the identification of pathogenic variants in *MT-TI*, *MT-TF*, *KCNJ16* and *ATP1A1*. These novel findings highlight the importance of cell metabolism and basolateral membrane potential for Na^+^ reabsorption in the DCT.

**Summary:**

Altogether, these findings extend the genetic spectrum of Gitelman-like electrolyte alterations. Genetic testing of patients with hypomagnesemia and hypokalemia should cover a panel of genes involved in Gitelman-like syndromes, including the mitochondrial genome.

## INTRODUCTION

Gitelman syndrome is a recessive salt-wasting disorder characterized by hypomagnesemia, hypokalemia, metabolic alkalosis, hypocalciuria and activation of the renin-angiotensin-aldosterone system (RAAS) [1,2]. Patients often present in late childhood or early adulthood with nonspecific symptoms, including muscle weakness, tetany, hypotension and fatigue [3,4]. Typical complaints may also include salt craving and thirst as a reflection of salt-wasting. Gitelman syndrome is not a benign condition and may cause chondrocalcinosis due to hypomagnesemia, prolonged QTc interval and arrhythmias due to hypokalemia, glucose intolerance and immunodeficiencies [5–8]. The disease was first described in 1966 by Hillel Gitelman as a subtype of Bartter syndrome [2]. However, typical Bartter symptoms such as polyhydramnios, hypercalciuria, nephrocalcinosis, failure to thrive and an antenatal presentation are rare in Gitelman syndrome. Indeed, genetic investigations in the 1990 s revealed that Bartter and Gitelman syndrome are separate clinical entities [9–13].

Classic Gitelman syndrome is caused by biallelic mutations in solute carrier 12 subtype 3 (*SLC12A3*) encoding the Na^+^-Cl^−^-co-transporter (NCC), which is exclusively expressed in the distal convoluted tubule (DCT) [13]. The NCC facilitates apical Na^+^ and Cl^−^ transport in the DCT and is the therapeutic target of thiazide diuretics. As a consequence of impaired NCC-mediated Na^+^ reabsorption in the DCT, the Na^+^ delivery to the collecting duct is increased. Accompanied by RAAS activation, the high Na^+^ delivery results in increased K^+^ secretion in the collecting duct explaining the hypokalemia in Gitelman patients. The metabolic alkalosis develops secondary to hypokalemia. The hypomagnesemia is less well understood (extensively reviewed in [14]), but it is generally thought that a reduced DCT mass is a major contributor to this defect [15]. However, human data supporting this hypothesis are scarce.

In recent years, several seminal discoveries have been made to resolve the missing heritability in Gitelman syndrome [16^▪^,^17^^▪▪^]. This review, therefore, provides an overview of all known genetic causes of Gitelman-like syndromes. The differences in clinical presentation, genetic inheritance and molecular disease mechanism will be discussed. 

**Box 1:**
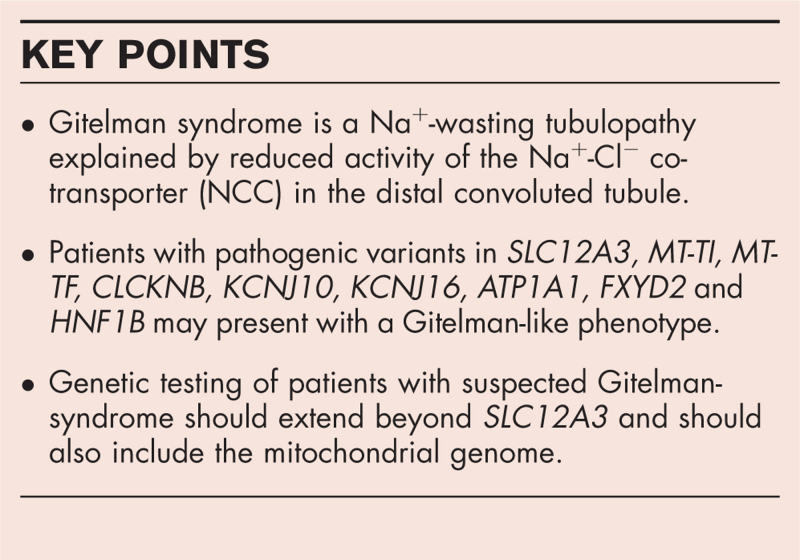
no caption available

## *SLC12A3* – CLASSIC GITELMAN

In 1996, Simon *et al*. [2] described homozygous and compound heterozygous loss-of-function mutations in *SLC12A3* as cause of Gitelman syndrome. Since then, 133 pathogenic variants have been described (ClinVar, February 2022), including deletions, splice site variants and intronic variants. In most recent screenings, approximately 75% of patients with a Gitelman syndrome presentation are diagnosed with a biallelic mutation in *SLC12A3*[18,19]. Of them, 20–25% have a homozygous pathogenic variant, 60–70% are compound heterozygous and ±10% have genomic rearrangements (deletion/duplication), which can be picked up by multiplex ligation-dependent probe amplification (MLPA) [18]. Homozygous mutations have been associated with an earlier age of onset and more severe hypocalciuria in a Chinese cohort [19]. In contrast, no phenotypic differences were reported for genomic rearrangements.

In-depth phenotyping of Gitelman patients with *SLC12A3* mutations has resulted in the identification of subclinical phenotypes [5,20^▪^]. In a large European cohort, 20% of patients with Gitelman syndrome had hypoparathyroidism [20^▪^]. As the parathyroid harmone (PTH) and magnesium concentrations were correlated in this cohort, it has been hypothesized that the hypoparathyroidism is explained by Mg^2+^-dependent regulation of the calcium-sensing receptor [21]. Alternatively, a positive Ca^2+^ balance may contribute to hypoparathyroidism in Gitelman syndrome. Several studies reported increased fasting glucose levels and insulin resistance in Gitelman patients [5,22,23]. In a large cohort of 77 patients, the insulin response was almost doubled upon glucose loading, which was associated with a significant increase of the insulin resistance index [5]. Indeed, diabetes mellitus has been reported in one-third of the patients in a Chinese cohort study [24]. Again, hypomagnesemia may (partially) explain the insulin resistance in Gitelman syndrome, as Mg^2+^ is essential for the insulin signalling pathway [25,26].

Interestingly, only one pathogenic variant is discovered in 10–15% of all Gitelman patients, even after screening for genomic rearrangements [18]. In these cases, mutations may be present in regulatory regions such as promoters and introns. Moreover, two patients were reported with a digenic inheritance pattern consisting of a heterozygous *SLC12A3* variant and a heterozygous *CLCKNB* variant [27,28]. However, it should be noted that it has not been conclusively demonstrated that digenic inheritance can cause Gitelman syndrome. Given that 2–8% of the population are carriers of one pathogenic *SLC12A3* variant and the percentage of carriers of one pathogenic *CLCKNB* variant may be similar, many patients should be affected by such an inheritance pattern [29].

**FIGURE 1:**
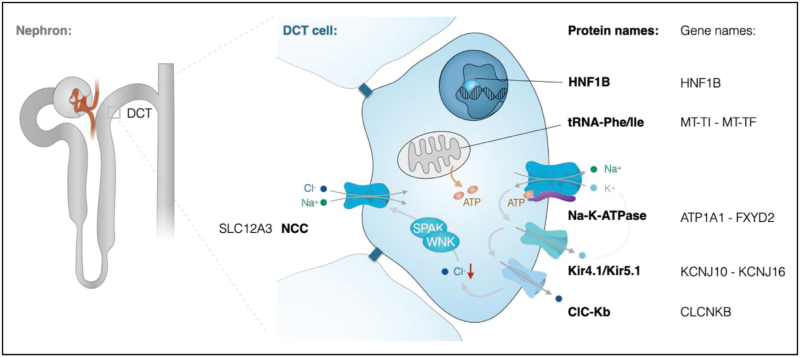
Na^+^ reabsorption in the distal convoluted tubule. Schematic overview of a distal convoluted tubule cell indicating all genes and proteins that have been associated with Gitelman syndrome. Na^+^ enters the cell at the luminal membrane via the Na^+^-Cl^−^ co-transporter (NCC). At the basolateral membrane, Na^+^ is extruded from the cell by the Na^+^-K^+^ ATPase. The ATP production required for Na^+^-K^+^ ATPase activity is dependent on mitochondrial function. Basolateral recycling of K^+^ via Kir4.1/Kir5.1 channels is essential to drive the Na^+^-K^+^ ATPase and Cl-extrusion via ClC-Kb Cl^−^ channels. Low intracellular Cl^−^ concentrations activate an intracellular signalling cascade of WNK and SPAK kinases, which results in phosphorylation of NCC.

Carriers of a single heterozygous pathogenic variant in *SLC12A3* were longtime considered healthy. However, recent studies have demonstrated the presence of a subclinical phenotype in heterozygous carriers [5,30^▪▪^]. Plasma aldosterone was slightly increased in carriers of heterozygous pathogenic *SLC12A3* variants [5]. Moreover, heterozygous carriers exhibited a slightly higher plasma Ca^2+^ concentration and lower plasma PTH concentration compared with controls. A recent study in the Old Order Amish population demonstrated that heterozygous carriers of the pathogenic p.R642G variant had significantly lower serum potassium levels than noncarriers [30^▪▪^]. These clinical findings are in line with mechanistic studies demonstrating the close connection of NCC and K^+^ regulation, termed the ‘potassium switch’ [31]. In short, the potassium switch turns on NCC in response to low dietary K^+^ intake and off in response to high K^+^ intake (Fig. 1) [32,33]. As such, K^+^ is currently considered as the main regulator of NCC activity, acting as a natural thiazide diuretic [34].

Altogether, these studies demonstrate that common genetic variants and heterozygous pathogenic variants in *SLC12A3* may contribute to subclinical phenotypes in the general population.

## *MT-TI / MT-TF* – MITOCHONDRIAL GITELMAN

In 2004, Lifton *et al*. [35] first described mutations in the mitochondrial transfer RNA (tRNA) for isoleucine, encoded by the *MT-TI* gene, in a large family with renal hypomagnesemia, hypokalemia and hypocalciuria. Only recently, these findings were confirmed in 10 additional families with a maternal inheritance pattern [17^▪▪^]. A large European collaboration demonstrated that mitochondrial DNA variants in *MT-TI* and *MT-TF* are causative for a Gitelman-like syndrome [17^▪▪^]. Interestingly, the *MT-TF* mutations were also associated with chronic kidney disease, whereas patients with *MT-TI* mutations showed a preserved kidney function [17^▪▪^]. Hypertension and dyslipidemia that were originally described to be part of the phenotype were not reported in these additional families, questioning whether this initial association was correct.

The identification of mitochondrial DNA mutations demonstrated the essential role of mitochondria for renal Na^+^ reabsorption. The DCT cell is the most mitochondria-rich cell type within the kidney in order to meet the high energy demand required for electrolyte transport [36]. In patient fibroblasts, the identified *MT-TI* and *MT-TF* mutations were demonstrated to reduce mitochondrial function [17^▪▪^]. Although the exact mechanisms remain unclear, pharmacological inhibition of complex IV, mimicking the effect of the mtDNA variants, inhibited NCC phosphorylation and NCC-mediated Na^+^ uptake [17^▪▪^]. However, it should be noted that only specific *MT-TI* and *MT-TF* mutations are associated with a Gitelman-like phenotype. Particularly, the m.591C>T and m.4291T>C variants are hotspot mutations. Other *MT-TI* and *MT-TF* mutations also resulting in reduced mitochondrial function have been associated with other syndromes such as mitochondrial encephalomyopathy, lactic acidosis and stroke-like episodes (MELAS) and myoclonic epilepsy with ragged-red fibres (MERRF) [37]. Consequently, one may consider additional pathophysiological mechanisms such as disturbances in tRNA modifications or effects of mitochondrial DNA fragments [38,39].

## *CLCNKB* – BARTTER TYPE 3

Although recessive *CLCKNB* mutations have originally been described to cause classic Bartter syndrome (type 3), a systematic analysis of a large cohort of patients demonstrates that 25% of all patients present with a Gitelman syndrome phenotype [10,40,41]. In fact, some patients may initially show symptoms of Bartter syndrome and develop a typical Gitelman phenotype in later childhood or adolescence [42]. Consequently, genetic screening of patients with a clinical diagnosis of Gitelman syndrome quite regularly turn out to have *CLCKNB* mutations upon genetic screening [43,44]. As large deletions account for up to 40% of all cases of Bartter syndrome type 3, testing for structural variations by MLPA or other means is advised [41]. Compared with classic Gitelman syndrome, patients with *CLCKNB* mutations have generally an earlier age of initial presentations and slightly higher serum Mg^2+^ and urinary Ca^2+^ concentrations [45,46]. Patients with *CLCKNB* mutations may additionally develop chronic kidney disease (up to 25%), nephrocalcinosis (10–20%) or growth retardation [41,46].

*CLCKNB* encodes the ClC_Kb_ Cl^−^ channel that is expressed in the TAL, DCT and collecting duct. Loss-of-function mutations in ClC_Kb_ result in an increased intracellular Cl^−^ concentration. As Cl^−^ inhibits WNK kinases, an increased Cl^−^ concentration causes reduced NCC activity by inhibition of the WNK-SPAK/OSR1 pathway (Fig. 1) [34,47,48]. A similar regulatory mechanism of NKCC2 exists in the TAL, which explains why ClC_Kb_ mutations may result in both Bartter-like and Gitelman-like syndromes [49]. In general, hypochloremia and increased fractional excretions of Na^+^ and Cl^−^ are more severe in Bartter syndrome type 3 than in Gitelman syndrome, which may reflect that both TAL and DCT are affected by *CLCKNB* mutations [45].

## *KCNJ10/ KCNJ16* – EAST / SESAME

The acronym EAST/SeSAME syndrome describes a disease entity with autosomal recessive inheritance combining epilepsy, ataxia, sensorineural deafness and renal tubulopathy with/without mental retardation [50,51]. Patients usually present early in infancy with seizures, developmental delay and ataxia. The renal phenotype closely resembles Gitelman syndrome comprising hypokalemic alkalosis, hypomagnesemia and hypocalciuria. EAST/SeSAME syndrome is caused by loss-of-function mutations in the *KCNJ10* gene encoding the inwardly rectifying K^+^-channel Kir4.1 [50,51]. In the kidney, Kir4.1 is predominantly expressed at the basolateral membrane of cTAL, DCT and CNT cells. Here, it forms heteromers with its close homologue Kir5.1 (*KCNJ16*). Kir4.1/Kir5.1 potassium channels serve as a K^+^ sensor of DCT cells [14,34] that allow for a recycling of K^+^ to drive Na^+^-K^+^-ATPase activity [32,52]. Uncoupling of this ‘pump-leak mechanisms’ will result in depolarization of the basolateral membrane and increased intracellular Cl^−^ concentrations, similar to mutations in ClC_Kb_ (Fig. 1) [10]. Consequently, the WNK-SPAK/OSR1 signalling cascade is inhibited resulting in reduced NCC activity.

Recently, recessive loss-of-function mutations have also been described in *KCNJ16* leading to a tubulopathy with deafness [16^▪^,^53^]. Apart from renal salt wasting and hypokalemia, patients may present with opposite changes in acid-base metabolism that are thought to result from a broader expression pattern and more diverse tasks of Kir5.1: In addition to its role in the DCT outlined above, Kir5.1 also forms heteromers with Kir4.2 (*KCNJ15*) in the proximal tubule that are critical for bicarbonate reabsorption and ammonia excretion [54]. However, if distal tubular salt wasting predominates, patients with *KCNJ16* mutations may present with hypokalemic alkalosis and a Gitelman syndrome-like phenotype [16^▪^].

## *ATP1A1/ FXYD2* – NA^+^-K^+^-ATPASE DYSFUNCTION

More than two decades ago, a missense mutation in *FXYD2* encoding the γ-subunit of Na^+^K^+^-ATPase was described in two related families. The index patients presented with seizures during childhood and profound hypomagnesemia [55,56]. Laboratory investigations revealed low serum Mg^2+^ levels also in numerous, apparently healthy family members. In addition, urinary Ca^2+^ excretion rates were found to be low, a finding reminiscent of patients presenting with Gitelman Syndrome. Later, a careful biochemical workup in members of two additional families with the identical mutation also revealed a tendency towards hypokalemia and metabolic alkalosis. Additional clinical findings in affected members of these families comprised muscle cramps, seizures and chondrocalcinosis [55–57].

Members of the FXYD protein family constitute a third, tissue-specific γ-subunit of Na^+^-K^+^-ATPase. *FXYD2* is expressed in the distal nephron, especially in the DCT and connecting tubule [58]. Here, the *FXYD2* γ-subunit increases the apparent affinity of Na^+^-K^+^-ATPase for ATP while decreasing its Na^+^ affinity providing a mechanism for balancing energy utilization and maintaining appropriate salt gradients [59]. Expression studies of mutant p.G41R-*FXYD2* revealed a dominant-negative effect leading to a retention of the γ-subunit in the Golgi complex [60].

Recently, also heterozygous de-novo mutations in the α1-subunit of Na^+^K^+^-ATPase (*ATP1A1*) have been described leading to severe hypomagnesemia due to renal magnesium wasting [61]. Affected children presented in infancy with seizures that were not responsive to antiepileptic medication and did not respond to magnesium supplementation. Unfortunately, all three children developed a significant degree of mental retardation and global developmental delay. In addition, episodes of hypokalemia and elevated bicarbonate levels potentially indicated renal salt wasting [61].

The α1-subunit *ATP1A1* represents the exclusive α-subunit of Na^+^K^+^-ATPase in kidney. Here, the DCT represents the tubular segment with the highest energy consumption and density of Na^+^K^+^-ATPase that generates a favourable electrochemical gradient for transcellular salt and magnesium reabsorption. Moreover, the α1-subunit is ubiquitiously expressed and thought to maintain neuronal housekeeping functions in the central nervous system [62]. The *ATP1A1* mutations discovered in hypomagnesemic children were shown to not only lead to a loss of ATPase function, but also to result in abnormal ion permeabilities and leak currents [61].

Whereas in children with *ATP1A1* mutations, the severe neurological phenotype is clearly distinct from GS; both entities, *FXYD2* and *ATP1A1*, share a renal GS-like phenotype even though a profound renal magnesium loss prevails.

## *HNF1B* – ADTKD-HNF1B

Hypomagnesemia and hypocalciuria are common in patients with heterozygous HNF1β mutations and deletions [63–66]. In a minor group of patients, these electrolyte disturbances are accompanied by hypokalemia and metabolic alkalosis [67,68] (Table 1). In addition, patients with HNF1β nephropathy often present with symptoms beyond a Gitelman-like phenotype including, but not limited to, tubule interstitial kidney disease (ADTKD), renal cysts, renal hypoplasia, hyperuricemia, hyperparathyroidism, maturity-onset diabetes of the young (MODY5), neurodevelopmental disorders, or genital and urinary tract malformations [64,69–72]. Approximately 50% of ADTKD-HNF1β patients develop chronic kidney disease [67,71,73]. HNF1β defects are therefore among the most common causes of childhood kidney transplantation [74,75]. Interestingly, in some cases, the electrolyte disturbances might represent the first symptom of the disease [63]. Consequently, the initial diagnosis of HNF1β nephropathy has sometimes been Gitelman syndrome, until genetic investigations revealed mutations in the *HNF1β* gene [43]. Of note, renin-angiotensin-aldosterone system (RAAS) activation is scarce in patients with HNF1β defects, whereas it is one of the main symptoms of Gitelman syndrome. Moreover, hypertension is present in 22% of children with HNF1β nephropathy [76]. Gitelman patients are generally hypotensive compared with healthy family members, though cases with hypertension in later life have been described [6,77]. Several reports noted that young children with HNF1β defects have generally higher serum Mg^2+^ levels than older patients [63,68,72]. It has, therefore, been proposed that hypomagnesemia developed later in childhood. However, Kolbuc *et al*. [65] recently showed that serum Mg^2+^ levels are also higher in early childhood of healthy controls. Consequently, the reference range of 0.7–1.1 mmol/l may not be suitable for young children, resulting in an underestimation of hypomagnesemia in early childhood of ADTKD-HNF1β patients.

**Table 1:** Overview of Gitelman(-like) sydromes

Gene	Protein	Disease	OMIM	Inh.	Onset	Mg^2+^	K^+^	HCO_3_^−^	FECa^2+^	RAAS	Other symptoms	Ref
*SLC12A3*	NCC	Classic Gitelman syndrome	263800	R	Childhood Adolescence	↓	↓	↑	↓	↑	Chondrocalcinosis	[1,2,13,46]
*MT-TI*	Mitochondrial tRNA-Ile	Mitochondrial Gitelman syndrome		M	Adult	↓	↓	=/↑	↓	=/↑		[17^▪▪^,^35^]
*MT-TF*	Mitochondrial tRNA-Phe	Mitochondrial Gitelman syndrome		M	Childhood Adult	↓	↓	=/↑	↓	=/↑	CKD	[17^▪▪^]
*CLCNKB*	ClC_Kb_	Bartter syndrome type III	607364	R	Neonatal Childhood	↓/=	↓	↑	↓/=/↑	↑	CKD	[10,45,46]
*KCNJ10*	Kir4.1	SESAME / EAST syndrome	612780	R	Neonatal	↓	↓	↑	↓	↑	Epilepsy, ataxia, sensorineural deafness	[50,51]
*KCNJ16*	Kir5.1		619406	R		↓/=	↓	↓/↑	↓	↑	Deafness	[16^▪^,^53^]
*FXYD2*	γ-subunit of the Na^+^-K^+^-ATPase		154020	D	Childhood Adult	↓	</=	=/↑	↓		Chondrocalcinosis	[56,57]
*ATP1A1*	α-subunit of the Na^+^-K^+^-ATPase		618314	D	Neonatal	↓	↓/=	=	↓/=/↑	=	Intellectual disability	[61]
*HNF1B*	HNF1β	ADTKD-HNF1B	137920	D	Neonatal Childhood	↓/=	=	=	↓	=/↑	CAKUT MODY5	[66,68]

In the DCT, HNF1β acts a transcription factor that regulates the expression of several proteins in the regulatory pathway towards NCC, including *FXYD2* and *KCNJ16*[69,78,79]. Potassium channel Kir4.1/Kir5.1 and the Na^+^-K^+^-ATPase activity are both essential components of the ‘pump-leak mechanism’ regulating the membrane potential and basolateral Cl^−^ transport. Disturbed transcription of *FXYD2* and *KCNJ16* thereby results in reduced NCC activity by the same mechanisms as described above. Clinical studies confirmed that ADTKD-HNF1β patients have reduced NCC activity, as indicated by a diminished response to thiazide [80]. In line with these findings, NCC expression is decreased in kidney-specific HNF1β knock-out mice [79].

## OTHER GENES

Several other non-Bartter, non-Gitelman syndromes are associated with salt-wasting, hypomagnesemia and hypokalemic alkalosis. Although these syndromes are independent of NCC dysfunction and therefore do not present as classical Gitelman syndrome, the presentation of individual patient may sometimes be, at least partially, similar.

Hypomagnesemia, hypokalemia and metabolic alkalosis are the cardinal symptoms of patients with mTOR-activating mutations in *RRAGD*, encoding a small Rag GTPase [81]. These patients often present with nephrocalcinosis and/or cardiomyopathy. As this disorder is often associated with renal Ca^2+^ wasting, it is hypothesized that *RRAGD* mutations primarily cause a defect in the TAL [81]. However, DCT defects cannot be excluded as *RRAGD* is also expressed in this segment of the nephron [81].

Impaired transcellular transport in the TAL is also the cause of salt-wasting in patient with *CLDN10* mutations. Patients suffer from hypokalemic hypochloremic alkalosis and RAAS activation, but generally present with hypermagnesemia [82,83]. Additional symptoms of *CLDN10* patients include dysfunctional salivary, sweat and lacrimal glands [83].

Hypomagnesemia is frequently associated with hypokalemia. This effect is generally explained by the inhibitory effect of Mg^2+^ on ROMK-mediated K^+^ secretion in the distal nephron [84]. In case of Mg^2+^ deficiency, more K^+^ is wasted in the urine resulting in hypokalemia. Genetic syndromes of isolated hypomagnesemia, for example by mutations in *TRPM6*, *KCNA1*, *EGF*, *CNNM2* or *PCBD1* may therefore present with transient episodes of hypokalemia [85–91]. However, these patients are generally without metabolic alkalosis or RAAS activation.

## NONGENETIC CAUSES OF GITELMAN-LIKE ELECTROLYTE ABNORMALITIES

Although it goes beyond the scope of this review to discuss all noninherited conditions that can mimic the presentation of Gitelman syndrome, it is important to consider alternative causes of Gitelman syndrome in clinical practice. In particular, abuse of diuretics (most notably thiazides) may result in an identical presentation [92,93]. In addition, chronic use of proton-pump inhibitors, aminoglycosides or laxatives is accompanied by hypokalemia and hypomagnesemia, although metabolic alkalosis is generally absent [92]. Other causes of hypokalemia may include chronic vomiting and primary hyperaldosteronism, but the latter condition is associated with hypertension and a suppressed RAAS [94]. Further guidance on the clinical workup and treatment of Gitelman syndrome is provided by KDIGO [1].

## CONCLUSION AND PERSPECTIVES

The discovery of *SLC12A3* mutations in the 1990 s established a defective salt reabsorption in the DCT as the underlying pathophysiology of Gitelman syndrome. Genetic heterogeneity of Gitelman syndrome was first demonstrated by the discovery of *CLCNKB* mutations in patients with a typical Gitelman syndrome-like phenotype. Since then, advances in genetics have led to the discovery of a growing number of hereditary disorders that present with the pathognomonic Gitelman syndrome signature comprising hypokalemic alkalosis, hypomagnesemia and hypocalciuria. Beyond representing important differential diagnoses for the molecular screening of affected patients, these entities not only underline the complex integrative role, but also vulnerability of the Na^+^ reabsorption machinery in the DCT. Here, transport processes are particularly dependent on cellular electrolyte homeostasis, energy level, respiratory capacity and regulatory pathways. This hereditary and phenotypic complexity will have to be taken into account by NGS-based analytic techniques as well as genetic counselling of the affected families. It appears reasonable to assume that future genetic studies will further expand the spectrum of disorders leading to defective DCT-mediated salt reabsorption or exhibiting the Gitelman syndrome-triad of hypokalemic alkalosis, hypomagnesemia and hypocalciuria as part of a more complex phenotype.

## Acknowledgements

*None.*

### Financial support and sponsorship

*This work was financially supported by ZonMW under the frame of EJPRD, the European Joint Programme on Rare Diseases (EJPRD2019–40) and by the IMAGEN project, which is co-funded by the PPP Allowance made available by Health∼Holland, Top Sector Life Sciences & Health, to stimulate public-private partnerships (IMplementation of Advancements in GENetic Kidney Disease, LSHM20009) and the Dutch Kidney Foundation (20OP+018). In addition, this project has received funding from the European Union's Horizon 2020 research and innovation programme under the EJP RD COFUND-EJP No. 825575 and the European Research Council (IN-THE-KIDNEY No. 101040682).*

### Conflicts of interest

*There are no conflicts of interest.*

## REFERENCES AND RECOMMENDED READING

Papers of particular interest, published within the annual period of review, have been highlighted as:

▪ of special interest
▪▪ of outstanding interest
